# Early structural valve deterioration of balloon expandable transcatheter aortic valve leaflets due to intrinsic and extrinsic nodular calcifications in a haemodialysis patient: a case report

**DOI:** 10.1093/ehjcr/ytae265

**Published:** 2024-05-24

**Authors:** Kyohei Onishi, Kazuki Mizutani, Yu Sato, Gaku Nakazawa

**Affiliations:** Division of Cardiology, Department of Medicine, Kindai University Faculty of Medicine, 377-2 Ohno-Higashi, Osakasayama, Osaka 589-8511, Japan; Division of Cardiology, Department of Medicine, Kindai University Faculty of Medicine, 377-2 Ohno-Higashi, Osakasayama, Osaka 589-8511, Japan; Department of Cardiology, Tokai University School of Medicine, Isehara, Japan; Division of Cardiology, Department of Medicine, Kindai University Faculty of Medicine, 377-2 Ohno-Higashi, Osakasayama, Osaka 589-8511, Japan

**Keywords:** Aortic valve stenosis, End-stage renal disease, Structural valve deterioration, Case report

## Abstract

**Background:**

Several reports have shown that transcatheter aortic valves are comparable in durability to surgical aortic valves. However, early structural valve deterioration (SVD) is rarely reported to occur, especially in haemodialysis patients.

**Case summary:**

We present a case of rapidly progressive bioprosthetic aortic valve stenosis in a patient with end-stage renal disease secondary to diabetic nephropathy in an 83-year-old female admitted due to progressive dyspnoea and orthopnoea. A 23 mm sized SAPIEN3 bioprosthetic aortic valve showed normal function for the first year after transcatheter aortic valve implantation (TAVI), but then rapidly developed stenosis and required acute hospitalization for heart failure a year and a half after surgery. Emergent surgical aortic valve replacement with a 19 mm On-X valve (CryoLife, Kennesaw, GA, USA) was performed. Pathological examination of the explanted SAPIEN 3 valve demonstrated severely degenerated bioprosthetic pericardial leaflets with severe intrinsic and extrinsic nodular calcifications, which could limit the leaflet motion.

**Discussion:**

There is a lack of reports on the long-term procedural outcomes of TAVI in haemodialysis patients. The development of SVD in patients undergoing dialysis is multifactorial and has yet to be fully elucidated. In the presented case, the removed TAVI valve had severe extrinsic calcified nodules alongside a fibrin thrombus. Considering these pathological findings, antithrombotic therapy to prevent fibrin thrombus from adhering to the TAVI valve may be important to avoid early SVD.

**Learning points**

- Although the therapeutic efficacy of transcatheter aortic valve implantation (TAVI) and the long-term durability of the bioprosthetic valve are comparable to those of surgical aortic valve replacement, early structural valve deterioration may rarely be experienced especially in end-stage renal disease (ESRD) patients.
- More closer echocardiographic follow-up is needed in ESRD patients who underwent TAVI, and appropriate antithrombotic therapy should be selected when the possibility of a thrombus valve is suggested.

## Introduction

Transcatheter aortic valve implantation (TAVI) has been recognized as a valid therapeutic option for patients with any surgical risk, and many young patients with aortic stenosis now undergo TAVI.^1–3^ Furthermore, the durability of TAVI bioprosthetic valves has been reported to be non-inferior to surgical bioprosthetic valves at 8 years post-operative data.^4^ However, early post-operative structural valve deterioration (SVD) of biological valves has been reported both after TAVI and surgical aortic valve replacement (SAVR), with a particularly high incidence in patients with end-stage renal disease (ESRD). We report a case of early SVD after TAVI in a patient with ESRD who underwent emergent SAVR with a mechanical valve and describe the mechanism of SVD development from pathological findings in the retrieved TAVI valve.

## Summary figure

**Table:** 

Time	Event
Two and a half years before admission	Started on haemodialysis. (ESRD due to diabetic nephropathy)
One and half years before admission	Transcatheter aortic valve replacement with a 23 mm SAPIEN3 (Edwards Lifesciences, Irvine, CA, USA) for severe aortic valve stenosis.
A half years before admission	No evidence of prosthetic valve dysfunction, with a mean aortic valve pressure gradient (mAVPG) of 18 mmHg and an EOA of 1.82 cm^2^ at the one-year follow-up.
One month before admission	Presented with dyspnoea and orthopnoea.
Time of admission	The patient was admitted to our institution because of acute heart failure.
Day 6 after admission	Emergency surgical aortic valve replacement was performed with a 19 mm On-X valve (CryoLife, Kennesaw, GA, USA).
Day 11 after admission	Move to the general ward.
Day 25 after admission	Changed to a rehabilitation hospital. A mAVPG of 14 mmHg and an EOA of 1.84 cm^2^ at discharge.

## Case presentation

An 84-year-old woman was admitted to our institution because of rapidly progressive dyspnoea and orthopnoea over the past month despite aggressive dehydration during outpatient dialysis.

She had a history of ESRD due to diabetic nephropathy and had been on haemodialysis for two and a half years. Additionally, she had a history of dyslipidaemia and hypertension. One and a half years prior, she began experiencing chest discomfort and shortness of breath on exertion. A Levine three-sixths degree systolic ejection murmur and a coarse crackle were heard from her. Electrocardiogram showed sinus rhythm, high voltage, and strain T pattern in chest leads suggesting left ventricular hypertrophy. Chest X-ray revealed cardiomegaly and pulmonary congestion, and serum brain natriuretic peptide level was 1290 pg/mL (normal range ≦ 18.4 pg/mL). Echocardiography revealed severe AS with a mean aortic valve pressure gradient (mAVPG) of 41 mmHg and an aortic valve area of 0.72 mm^2^. She underwent TAVI with a 23 mm SAPIEN3 (Edwards Lifesciences) a year and a half prior to this admission. Following the procedure, the patient’s recovery was uneventful, and echocardiography showed no paravalvular leak, a mAVPG of 17 mmHg, and an effective orifice area (EOA) of 1.92 cm^2^ at discharge (see Supplementary  *Movie S1*). She had been receiving antiplatelet therapy with clopidogrel 75 mg after TAVI. Echocardiography at the one-year follow-up revealed no evidence of prosthetic valve dysfunction, with a mAVPG of 18 mmHg and an EOA of 1.82 cm^2^ (see Supplementary  *Movie S2*).

**Figure 1:**
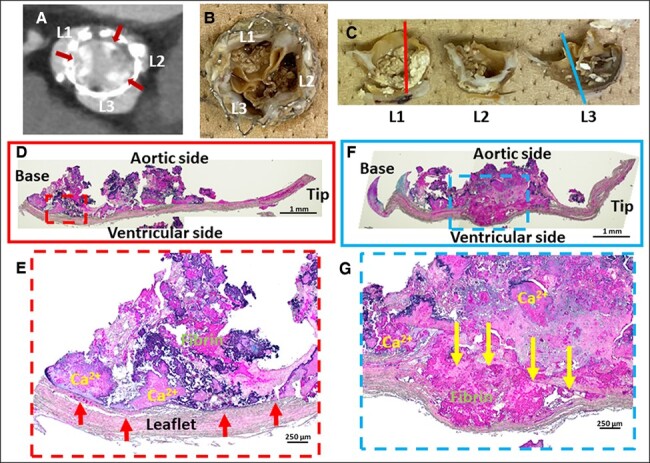
Contrast-enhanced computed tomography of the implanted TAVI valve (SAPIEN3) and representative histopathological images of the removed TAVI valve. Contrast-enhanced computed tomography showed severe calcification and thickening in all three prosthetic valve leaflets. (*A*) The removed TAVI valve showed adherence of nodular calcification in all tricuspids. (*B* and *C*) Gross images of explanted SAPIEN3 valve showing nodular calcification protruding to the aortic side of the leaflets. Low-power and high-power images of leaflet 1. (*D* and *E*) There is a severe leaflet extrinsic calcification containing calcific nodules and fibrin deposition. The surface of the leaflet is intact, and the border is easily recognized (red arrows). Low-power and high-power images of leaflet 3. (*F* and *G*) The leaflet is severely thickened and composed of intrinsic and extrinsic nodular calcifications. Yellow arrows indicate the separation of the leaflet collagen fibres, and intrinsic nodular calcification is observed between these fibres.

The patient underwent transthoracic echocardiography at admission, which revealed stiffening and complete loss of mobility of the aortic prosthetic valves (see Supplementary  *Movie S3*). This resulted in severe AS with a mAVPG of 68 mmHg and an EOA of 0.65 cm^2^. Contrast-enhanced computed tomography (CT) showed severe calcification and thickening of all three prosthetic valve leaflets (*Figure 1*).

The CT findings suggested a high risk of coronary artery occlusion if TAV in TAV was selected. Even in the case of a repeated SAVR with a bioprosthetic valve, there remained a concern about high surgical risk, with an STS score of 13.8% and a significant likelihood of early SVD. Following a rapid multidisciplinary team discussion, we performed an emergency SAVR with a 19 mm On-X valve (CryoLife). Pathological observation of the explanted SAPIEN 3 valves demonstrated severely degenerated bioprosthetic pericardial leaflets with severe intrinsic and extrinsic calcifications that could cause limitation of the leaflet motion (*Figure 1*). The patient did well post-operatively, and there were no other major complications. Post-operative echocardiography showed improvement in AV peak flow [2.6 m/s, mAVPG (14 mmHg], and EOA (1.84 cm^2^). No heart failure exacerbations, warfarin-induced bleeding events or prosthetic valve dysfunction were observed in the first year after SAVR.

## Discussion

The incidence of moderate or severe SVD is ∼8.97% at 5 years after TAVI, with no significant differences in the risk of developing SVD between TAVI and SAVR at the 5-year mark.^5^ Notably, self-expanding TAVI had a significantly larger EOA and lower mAVPG in echocardiograms than balloon-expandable TAV and SAVR (1.84 ± 0.54 vs. 1.51 ± 0.49 vs. 1.39 ± 0.45, *P* < 0.01; 7.42 ± 3.60 vs. 11.61 ± 6.65 vs. 11.33 ± 5.39, *P* < 0.01).^6^ However, reports are lacking on the long-term procedural outcomes of TAVI in haemodialysis patients. On the other hand, several factors have been reported to predispose patients to SVD. Tobias *et al*.^7^ demonstrated that patients with a small valve diameter, balloon-expandable valve, renal dysfunction, or no direct oral anticoagulant exposure at discharge are at an increased risk of SVD. Structural valve deterioration is often reported to occur earlier in haemodialysis patients, and the incidence of moderate or severe SVD after SAVR in patients undergoing dialysis is significantly higher than in non-dialysis patients during the follow-up period.^8^ In contrast, the INSPIRIS RESILIA aortic valve (Edwards Lifesciences) and the SAPIEN3 Ultra RESILIA TAVI valve (Edwards Lifesciences) with improved anti-calcification treatment, which is considered helpful for avoiding SVD, are now available.^9^ However, early SVD after SAVR using the INSPIRIS RESILIA valve has been reported in a patient undergoing dialysis.^10^

In our presented case, the patient rapidly developed severe SVD after TAVI. While SAPIEN3 Ultra RESILIA was considered in the TAV procedure by the Heart Team, SAVR with mechanical valves was chosen due to the anatomically high risk of coronary occlusion and the high risk of another early SVD complication. We investigated the mechanisms underlying early SVD progression in patients undergoing dialysis. Although the mechanism of SVD progression has not been fully elucidated in patients undergoing dialysis,^11,12^ aortic valve calcification is reportedly accelerated by ectopic calcium deposition due to increased calcium and phosphate products, secondary hyperparathyroidism, and excess vitamin D.^13^ In this case, these factors were properly adjusted, and pathological examination of the removed TAVI valve revealed severe extrinsic calcified nodule along with a fibrin thrombus.

A recent pathological evaluation suggested that not only intrinsic but also extrinsic calcification can affect leaflet durability^14^ because leaflet thrombus, which is thought to be seen as HALT in clinical CT, can calcify and cause extrinsic calcification.^15^ Our case suggests that antithrombotic therapy may effectively prevent SVD due to extrinsic calcification. On the other hand, intrinsic calcification tends to be seen in cases with longer duration.^14^ To our knowledge, the current case was the first case report that pathologically demonstrated SVD of the TAVI valve due to severe intrinsic and extrinsic nodular calcifications in a haemodialysis patient. Further studies are needed to investigate the optimal medical therapy in haemodialysis patients who received TAVI for aortic valve stenosis. Finally, I would like to emphasize that lifetime management is important in young AS patients with low surgical risk, and if TAVI is chosen, it is also necessary to evaluate whether TAV in TAV is feasible.^16^

## Supplementary Material

ytae265_Supplementary_Data

## Data Availability

Data sharing is not applicable to this article, as no datasets were generated or analysed during the current study. The data underlying this article are available in the article and online Supplementary material.

## Lead author biography

Kyohei Onishi graduated from Kindai University Hospital in 2015. He is working at Kindai University Hospital, Department of Cardiology. His speciality is ischaemic heart disease and structural heart disease.
